# Advancements in Detection Approaches of Severe Acute Respiratory Syndrome Coronavirus 2

**DOI:** 10.21315/mjms2022.29.6.3

**Published:** 2022-12-22

**Authors:** M. A. Motalib Hossain, Syed Muhammad Kamal Uddin, Abu Hashem, Mohammad Al Mamun, Suresh Sagadevan, Mohd Rafie Johan

**Affiliations:** 1 Nanotechnology and Catalysis Research Centre, Institute for Advanced Studies, Universiti Malaya, Kuala Lumpur, Malaysia; 2 Microbial Biotechnology Division, National Institute of Biotechnology, Dhaka, Bangladesh; 3 Department of Chemistry, Jagannath University, Dhaka, Bangladesh

**Keywords:** COVID-19, SARS-CoV-2, detection approach, nucleic acid, RT-qPCR

## Abstract

Diagnostic testing to identify individuals infected with severe acute respiratory syndrome coronavirus 2 (SARS-CoV-2) plays a key role in selecting appropriate treatments, saving people’s lives and preventing the global pandemic of COVID-19. By testing on a massive scale, some countries could successfully contain the disease spread. Since early viral detection may provide the best approach to curb the disease outbreak, the rapid and reliable detection of coronavirus (CoV) is therefore becoming increasingly important. Nucleic acid detection methods, especially real-time reverse transcription polymerase chain reaction (RT-PCR)-based assays are considered the gold standard for COVID-19 diagnostics. Some non-PCR-based molecular methods without thermocycler operation, such as isothermal nucleic acid amplification have been proved promising. Serologic immunoassays are also available. A variety of novel and improved methods based on biosensors, Clustered-Regularly Interspaced Short Palindromic Repeats (CRISPR) technology, lateral flow assay (LFA), microarray, aptamer etc. have also been developed. Several integrated, random-access, point-of-care (POC) molecular devices are rapidly emerging for quick and accurate detection of SARS-CoV-2 that can be used in the local hospitals and clinics. This review intends to summarize the currently available detection approaches of SARS-CoV-2, highlight gaps in existing diagnostic capacity, and propose potential solutions and thus may assist clinicians and researchers develop better technologies for rapid and authentic diagnosis of CoV infection.

## Introduction

A novel coronavirus, officially termed as severe acute respiratory syndrome coronavirus 2 (SARS-CoV-2), has been identified by the Chinese Centre for Disease Control and Prevention (China CDC). The disease named coronavirus disease 2019 (COVID-19) have spread unprecedently throughout the whole world claiming a huge number of lives every day. Although a large number of cases have spontaneously cured, some are developing various fatal complications leading to death. The primary cause of mortality due to this emerging coronavirus (CoV) seems to be acute respiratory distress syndrome (ARDS) (1) with the association of comorbidities that ultimately cause multiple organ failure leading to death (2). CoVs are classified into the family *Coronaviridae* (3). Taxonomical relationship of SARS-CoV-2 has been observed with the subgenus *Sarbecovirus* along with SARS-CoV and bat SARS-like CoVs (4). Given rapid human-to-human transmission, SARS-CoV-2 has become a global public health threat (5). Generally, SARS-CoV-2 has an incubation period of 2–7 days with spreading capability (from an infected individual to a non-infected one) during this incubation period (6). Since the emergence of SARS-CoV-2, several new strains with varying traits have been identified. Scientists and clinicians are trying relentlessly to minimise the death toll the world is facing.

Diagnostic testing to identify individuals infected with SARS-CoV-2 plays a key role in selecting appropriate treatments, saving people’s lives and preventing the global pandemic of COVID-19. The rapid and accurate detection of CoV is therefore becoming increasingly important to curb the pandemic. Until now, a variety of diagnostic methods are available to detect SARS-CoV-2 infection. Among them, polymerase chain reaction (PCR), has become a revolutionary tool for viral detection and gained increased acceptability given their high sensitivity and specificity. Antibodies generated in response to viral infection can be used for the identification of infected individuals. However, the use of antibody-based diagnostics is not suitable to detect active diseases. Notably, most of the diagnostic tools used nowadays are time-consuming requiring skilled personnel, but for diagnostic testing on a massive scale, there is an increasing demand for portable devices that offer efficient and rapid detection. Consequently, newer laboratory methods using several integrated, point-of-care (POC) molecular devices are currently applied for fast and accurate diagnosis of SARS-CoV-2 infections. In addition to different diagnostic kits, US Food and drug administration (USFDA) has already approved the home COVID-19 diagnostic kits with a home collection facility to enhance the overall testing capacity (7).

Until now, several reviews have been documented on the detection methods of SAR-CoV-2 (8–10). COVID-19 pandemic is a fast-moving situation and related research findings are continuously and rapidly updating. In this regard, we documented this review article that encompasses the recent advancements in diagnostic approaches of COVID-19 and highlights gaps in current diagnostic capacity as well as proposes potential solutions that may be useful to develop strategies to deal with the current pandemic.

### Virology

CoVs are a member of the subfamily *Orthocoronavirinae* in the family *Coronaviridae* and the Order *Nidovirales*. The CoV genome is the largest (26 kb–32 kb) among the known RNA viruses and is an enveloped, positive-sense, single-stranded RNA. The CoVs are divided into four groups such as α, β, γ and δ. The SARS-CoV, MERS-CoV and SARS-CoV-2 (causative agent of COVID-19) are under β-CoV class. It is known that α-CoV (HCoV-229E and NL63) and β-CoVs usually infect mammals whereas δ- and γ-CoV genera are responsible for infection in birds (11, 12).

**Figure 1:**
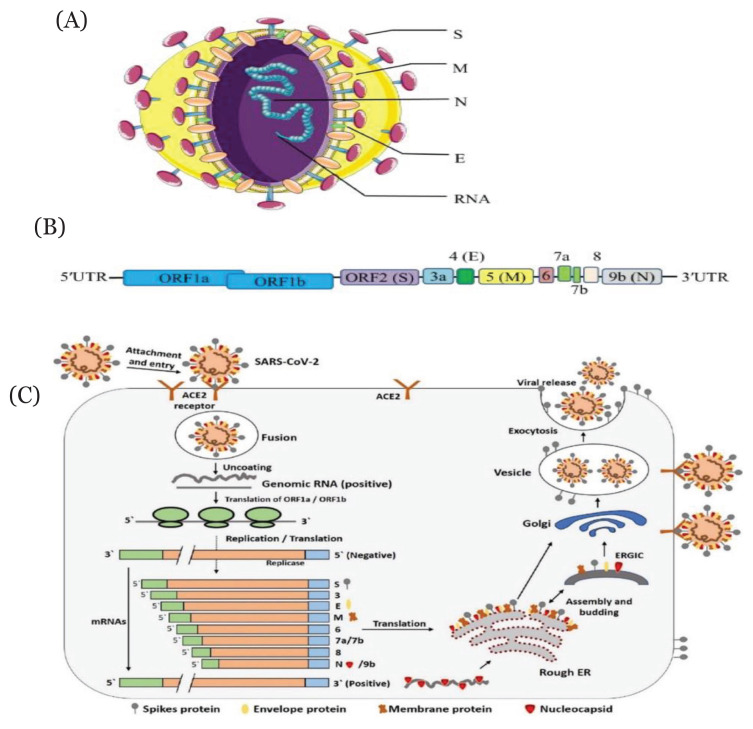
Structural presentation of SARS-CoV-2 (A and B) and schematic diagram of its life cycle in host cells (C). (A) Four structural proteins of SARS-COV-2 are as follows: spike (*S*) surface glycoprotein (as purple colour); membrane (*M*) protein (as orange colour); nucleocapsid (*N*) protein (as blue colour); and envelope (*E*) protein (as green colour). Genomic RNA has been represented as encased in the *N* protein. (B) The arrangement of SARS-CoV-2 genome is in the following order: 5′-replicase (ORF1a/b)–structural proteins [*S*–*E*–*M*–*N*]–3′ (reprinted from Li et al. (11) with permission). (C) Life cycle starts after binding of *S* protein to the cellular receptor ACE2. Following receptor binding, the *S* protein undergoes conformational change thus facilitating viral envelope fusion with the cell membrane through the endosomal pathway. Then RNA is released from SARS-CoV-2 into the host cell. Genome RNA is translated into viral replicase polyproteins pp1a and 1ab, which are then cleaved into small products by viral proteinases. A series of sub genomic mRNAs is produced by the polymerase through transcription and finally they are translated into relevant viral proteins. The resultant viral proteins and genome RNA are subsequently assembled into virions in the ER and Golgi and then transported via vesicles to be released out of the cell. Notes: ACE2 = angiotensin-converting enzyme 2; ER = endoplasmic reticulum; ERGIC = ER–Golgi intermediate compartment [reprinted from Shereen et al. (17) with permission]

A total of 27 proteins are encoded by the SARS-CoV-2 genome. Among these proteins, a large and non-structural polyprotein (ORF1ab) undergoes further proteolytic breakdown and generates 15–16 proteins including five accessory proteins (ORF3a, ORF6, ORF7, ORF8 and ORF9), four structural proteins and an RNA-dependent RNA polymerase (RdRP). RdRP, in combination with nonstructural proteins, maintains genome fidelity. The four structural proteins of SARS-CoV-2 such as spike (*S*) surface glycoprotein, the membrane (*M*) protein, the envelope (*E*) protein and the nucleocapsid (*N*) protein play an essential role in virus assembly and infection (11, 13). Among them, *S* protein is involved in recognition and binding to the surface receptors in host cells and mediates the fusion of virus envelop and cell membranes. The *M* protein is the most abundant structural protein which gives the shape of the viral envelope, promotes membrane curvature as well as is involved in the transportation of nutrients across cell membranes. Among the major structural proteins, the *E* protein is the smallest one which takes part in the assembly and release of virus (14–16, 17). The structure of SARS-COV-2 and its life cycle are illustrated in Figure 1.

### Detection Approaches of SARS-CoV-2

The initial step to control the spread of COVID-19 is to detect infection at a primary stage and instantly isolate infected patients from healthy individuals (18). Therefore, quick and reliable identification of SARS-CoV-2 is crucial to curb the outbreak of COVID-19. Currently available SARS-CoV-2 diagnostic approaches based on the recent research progress are summarised in Table 1.

### Nucleic Acid-based Detection Technology

Nucleic acid (NA) detection technique involves multiplication and detection of a segment of hereditary elements such as DNA or RNA. Quantitative reverse transcription PCR (RT-qPCR), isothermal NA amplification and high-throughput sequencing (HTS) are commonly practiced NA detection technologies for SARS-CoV-2.

### Quantitative Reverse Transcription PCR

The most acceptable diagnostic technique for the identification of SARS-CoV-2 is RT-qPCR. Herein, generally, specific gene fragments (RNA) of SARS-CoV-2 are initially reverse transcribed to cDNA followed by the amplification of target fragments of cDNA using specific primers and probes. Representative copy number of the target can be detected easily by the fluorescence signal generated during the amplification process (19, 20). For SARS-CoV-2 detection, several RT-qPCR primer sets have been designed (Table 2).

Within a short period after the epidemic of COVID-19, researchers have developed and launched RT-qPCR kits for medical diagnosis. Initially, for the detection of SARS-CoV-2 by RT-qPCR, the China CDC recommended the designed primers and probes specific to the ORF1ab and *N* gene regions. Chu et al. (21) developed two single-step RT-qPCR assays for the detection of two separate loci (ORF1b and *N*) of the virus genome. Targeting the nucleocapsid (*N*) genes, spike (*S*) genes and RdRP genes of SARS-CoV-2, three novel real-time RT-qPCR assays have been developed by Chan et al. (22). The efficiencies of their assays were also compared with the reported RdRP-P2 based assay, which has been applied in more than 30 laboratories in Europe. The COVID-19-RdRP assay, of the three developed assays, exhibited the lowest limit of detection (LOD) in vitro. The COVID-19-RdRP assay did not exhibit cross-reactivity with neither respiratory pathogens nor other pathogenic CoVs in clinical and cell culture samples but the RdRP-P2 assay showed cross-reactivity with SARS-CoV in cell culture specimens.

Corman et al. (23) designed primers and probes using three regions having conserved sequences within the SARS-related viral genomes: i) RdRP gene in ORF1ab region; ii) the envelope protein (*E*) gene and iii) the nucleocapsid protein (*N*) gene. After designing and testing, they found that both the RdRP and *E* genes showed high diagnostic sensitivity (LOD of 3.6 and 3.9 copies/reaction), while the *N* gene exhibited lower sensitivity (LOD 8.3 copies/reaction). Yip et al. (24) performed an in-house programme, GolayMetaMiner, using 96 SARS-CoV-2 and 104 non-SARS-CoV-2 genomes to identify highly conserved regions among globally detected SARS-CoV-2 isolates. They found 154 nt conserved regions in the nsp2 gene which was not present in other human infectious CoVs. Then, they designed the primer set from this conserved sequence and developed a SYBR Green-based highly sensitive and specific RT-qPCR assay with the LOD of 1.8 TCID_50_/mL. More recently, Smyrlaki et al. (25) reported rapid and direct single-reaction RT-qPCR method circumventing SARS-CoV-2 RNA-extraction on heat-inactivated nasopharyngeal swab samples. The proposed method is significant in saving both time and cost by eliminating the RNA-extraction and could help limit the spread of SARS-CoV-2.

As a robust and standard technique, RT-qPCR has been extensively used for the detection of SARS-CoV-2. However, it involves biological safety hazards during preservation and handling of infectious specimens, laborious NA diagnosis process and needs long analysis time (12). In addition, sample processing, laboratory settings and technical faults can sometimes lead to false-negative outcomes. Moreover, it requires experts and well-equipped laboratory set up that make its practical applications limited (6).

### Isothermal Nucleic Acid Amplification

Isothermal nucleic acid amplification is a one-tube and fixed temperature method for the amplification of NA and a cost-effective substitute for the diagnosis of certain diseases. Researchers are now applying this technique to detect NA for SARS-CoV-2. This process involves recombinase polymerase amplification, helicase-dependent amplification as well as loop-mediated isothermal amplification (LAMP). Reverse transcription LAMP (RT-LAMP) has been developed by some laboratories for the detection of SARS-CoV-2 (26–29). In LAMP assay, the amplified DNA is detected based on turbidity, colour change or fluorescence intensity (30).

Jiang et al. (31) developed a single-step RT-LAMP assay targeting nucleocapsid protein (N) region for fast and reliable detection of SARS-CoV-2. The developed method was compared with commercial RT-qPCR assays and was found to show a higher degree of sensitivity and specificity than RT-qPCR. Similarly, Park et al. (32) developed and assessed one-step RT-LAMP technique for detecting genomic RNA of SARS-CoV-2 and found LOD of 100 copies of this viral RNA without observing any cross-reaction with other counterparts of CoVs.

**Figure 2:**
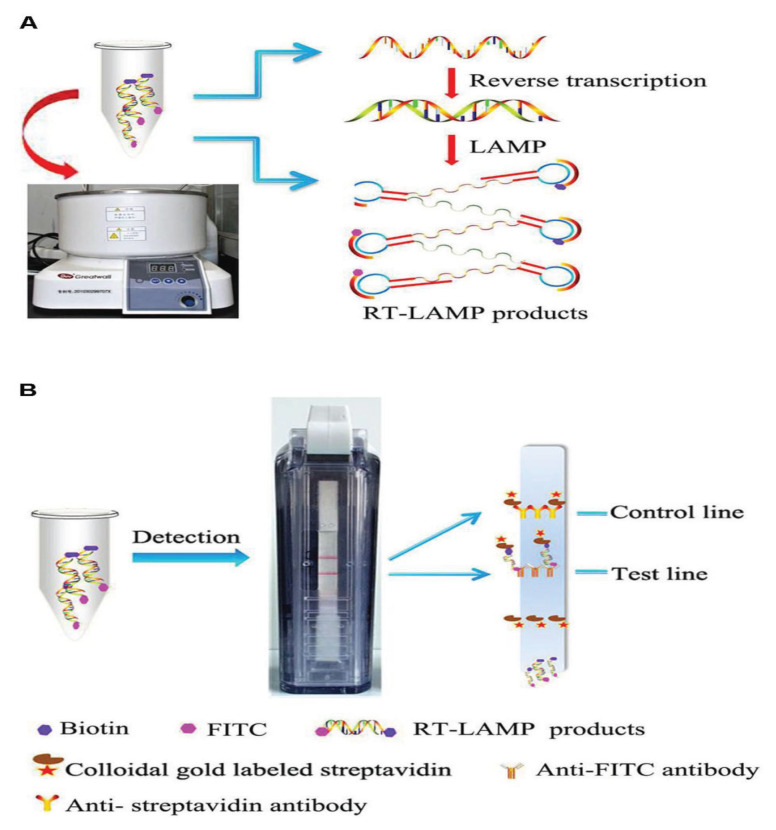
Schematic presentation of the RT-LAMP-VF assay: (A) Carrying out of RT-LAMP in a water bath maintaining constant temperature. (B) Detection of RT-LAMP products with a vertical flow visualisation strip [reprinted from Huang et al. (50) with permission]

Another group also designed specific primer sets based on *ORF1ab* and *S* genes sequences of SARS-CoV-2 to develop RT-LAMP technique. They optimised the RT-LAMP assay with the primer sets ORF1ab-4 and S-123 and amplified the target genes within a very short time of 18 ± 1.32 min and 20 ± 1.80 min, respectively having the optimal reaction temperature of 63 °C. The sensitivity was found 20 copies and 200 copies/reaction using primers ORF1ab-4 and S-123, respectively (33). Yang et al. (27) proposed a faster and more suitable RT-LAMP assay for SARS-CoV-2 where the genes ORF1ab, *E* and *N* were detected simultaneously. Another RT-LAMP was documented where magnetic bead virus genome capture and established RT-LAMP were combinedly applied targeting ORF1a gene for amplification and identification of the SARS-CoV-2 (34). In addition, Huang et al. (35) developed a NA visualisation assay that combines the RT-LAMP and a vertical flow visualisation strip (RTLAMP-VF) for detecting the *N* gene of MERS-CoV (Figure 2).

Lu et al. (36) developed a one-step single-tube RT-LAMP assay to detect SARS-CoV-2 using mismatch-tolerant LAMP technique where 0.15 U of high-fidelity DNA polymerase was used. The assay can be carried out at 63 ^0^C for 40 min using a conventional or real-time thermal cycler. The LOD of the assay was 30 copies per reaction. More recently, Thi et al. (37) reported a colorimetric RT-LAMP assay for detecting SARS-CoV-2 RNA targeting *N* gene.

RT-LAMP testing may be suitably used for SARS-CoV-2 detection even in those situations where sophisticated molecular biology instruments cannot be afforded. Nevertheless, the approach is newer and lacks enough research background behind it. Confirmatory diagnosis of COVID-19 using LAMP technology is still under assessment in clinical settings (38).

### Viral Genome Sequencing

Compared to traditional diagnosis methods, high throughput sequencing (HTS) is universal and more accurate for pathogen profiling (39). Besides confirming the existence of the virus, regular sequencing of a fraction of samples from clinical cases can be beneficial to observe viral genome alterations (mutations) that might influence the performance of diagnosis and treatment.

The most comprehensive method for the detection of viral NAs is whole-genome sequencing. Numerous data on complete genome sequences of SARS-CoV-2 are being uploaded by different research groups ( https://www.gisaid.org/). By using hybridisation capture technology, Zhang (40) developed a set of enrichment probes for SARS-CoV-2 to improve the sensitivity of sequence-based virus identification and characterisation. From one cultured strain of SARS-CoV-2 virus, they prepared six enrichment libraries to examine the effects of enrichment and sequenced those on MGI-2000 system. Liu et al. (6) developed a new technique namely nanopore target sequencing (NTS) for sequencing SARS-CoV-2 genome. At the preliminary step, this technique involves the amplification of some virulence gene fragments of SARS-CoV-2 followed by application of the nanopore platform to accomplish the amplified fragments sequencing. Thus, sequencing and analysis of results can be performed simultaneously in nanopore platform, which further allows confirmation of the existence of SARS-CoV-2 in a few minutes after sequencing.

The whole-genome sequencing is not appropriate for regular and large-scale testing and has not been extensively used as a useful analytical tool, as it is relatively costly, time consuming, requires expert professional personnel with expensive equipment settings as well as difficult to prepare sample and analyse data (6, 41).

### Serological Detection

Serological assays can be performed for disease diagnosis using the blood of an infected person. In response to an infection, viral proteins, antigens and antibodies are generated in patient’s blood and these can be used for disease diagnosis. Antibody diagnosis can be useful for the surveillance study of COVID-19. Serological assays can play a vital role to combat COVID-19 through detecting those persons who have recovered from infection and have developed an immune response together with other clinical records.

### Enzyme Linked Immunosorbent Assay

Enzyme linked immunosorbent assay *(*ELISA) is a lab-based serological test that may be either qualitative or quantitative. The test usually involves plates that are coated with target viral proteins. The patient sample is then incubated with that protein and if the patient carries antibodies to that particular protein, they bind together. The formed antibody-antigen composite can be recognised with the addition of second antibody that generates a colour or fluorescent-based reading. In case of COVID-19, this assay usually detects patient antibodies namely immunoglobulin G (IgG) and immunoglobulin M (IgM) (42).

Using an ELISA, Zhang et al. (43) detected IgG and IgM from COVID-19 infected patient’s serum. They used Rp3 nucleocapsid protein of SARS-CoV-2, which shows 90% amino acid sequence similarity with other SARS CoV relatives. After confirmation by RT-qPCR, they verified 16 SARS-CoV-2 infected patients’ samples with ELISA and observed that the levels of IgG and IgM have been elevated over the first 5 days after onset of symptoms. On day zero, 50% of patients were positive for IgM and 81%, for IgG, while these values raised to 81% and 100% at day 5, respectively. Guo et al. (44) proposed an ELISA method for SARS-CoV-2 detection using purified recombinant N proteins (rNPs) as coating antigens to detect IgA, IgM and IgG antibodies. The ELISA technique was also established based on rNP and recombinant spike protein (rSP) of SARS-CoV-2 for detection of IgM and IgG antibodies. In case of IgM recognition, the sensitivity of the rSP-based ELISA was substantially higher compared to rNP-based one (45). Li et al. (46) developed a Sandwich ELISA kit targeting SARS-CoV-2 rNP with the sensitivity of 100 ng/mL.

ELISA testing requires 1 h–5 h to provide information about the presence or absence of developed antibodies. However, these techniques, for SARS-CoV-2 detection, are yet to be applied on a large scale (38).

### Rapid Serological Tests

Many researchers and diagnostic kit producers have developed speedy and easy-to-apply tools to enable SARS-COV-2 detection beyond laboratory set-up. These user-friendly diagnostic kits are based on either identification of SARS-COV-2 viral antigens from the respiratory specimens like sputum, throat swab or detection of human serum antibodies produced in blood (47). These are typically qualitative tests (positive or negative) that require simple and portable devices that may be applied at POC.

### Lateral Flow Assay

Lateral flow assay (LFA) is one kind of user-friendly, cheap and easily developed POC approach that may be applied for in-field detection of SARS-CoV-2 (48). It is a paper-based technique that allows both detection and quantification of analytes in complex matrices displaying the result within a very short time. In a general format, a labeled antibody is allowed to bind with the unknown antigen. The resulting antigen-antibody complex then migrates under the influence of capillary action through the support. Then, if the antigen is present, a second antibody embedded in the solid support detects the complexes (49). LFA may be developed by incorporating nucleic acid testing. In a study, a RT-LAMP technique was combined with lateral flow readout for the detection of MERS-CoV (35). However, LFA suffers from poor detection sensitivity as compared to RT-qPCR and thus less suitable for accurate screening of COVID-19 patients. To improve the assay sensitivity, researchers have developed a variety of novel nanomaterials as immunolabels such as quantum dots, magnetic nanoparticles and up-conversion nanoparticles (50). By using ultra-bright fluorescence nanomaterials having longer fluorescence lifetimes, background noise can be considerably decreased and LFA detection sensitivity can be enhanced using the time-resolved analysis technique (51).

For simultaneous detection of IgM and IgG antibodies generated against SARS-CoV-2 in blood, Li et al. (52) established a fast and simple POC lateral flow immunoassay which is capable to detect different stages of infection in patients within 15 min. The overall detection sensitivity and specificity were 88.66% and 90.63%, respectively. The combined IgM and IgG detection showed better applicability and sensitivity compared to those obtained from single IgM or IgG detection. Grant et al. (53) reported a half-strip LFA by utilising commercial antibodies for detecting nucleocapsid protein of SARS-CoV-2. The LOD of the assay was found to be 0.65 ng/mL. Recently, Peng et al. (54) documented gold nanoparticle (AuNP) based LFA test kits for quantification of SARS-CoV-2 antibodies. Herein, to increase the assay sensitivity, the researchers improved the readout of the device and thus could quantify the antibody with higher sensitivity.

### Biosensor-based Detection

The conventional technique like RT-qPCR for detecting SARS-CoV-2 is performed in established laboratory. It is time-consuming, laborious, expensive and requires that all the specimens must be transported to the central laboratories (55). In this perspective, biosensor-based techniques might be the suitable alternative that may offer POC detection and real-time analysis.

A biosensor based on NA consists of a single-stranded oligonucleotide (either ssDNA or ssRNA) as a bio-sensing component immobilised on a signal-transducing surface for the detection of its complementary DNA (cDNA) or RNA sequence via hybridisation. As a result of hybridisation on the sensor surface, the formed hybrid is then transformed into an analytical signal via a signal transducer. The electrochemically designed NA biosensors are highly promising because they offer several potential benefits including portability, user-friendliness, low-cost, rapid response, remarkable sensitivity with selectivity and compatibility with miniaturised diagnostic devices (55).

Qiu et al. (56) have developed a plasmonic biosensor that possesses dual functionality. They have combined the plasmonic photothermal (PPT) effect with localised surface plasmon resonance (LSPR) sensing transduction offering an alternative and innovative approach for the diagnosis of COVID-19. The 2-D gold nanoislands (AuNIs) conjugated with cDNA as receptors can sensitively detect the specific NA sequences from SARS-CoV-2 via the hybridisation of nucleic acid. The developed dual-functional LSPR biosensor exhibited a higher sensitivity toward the selected SARS-CoV-2 sequences with a lower LOD of 0.22 pM.

In addition to NA based biosensors, researchers also developed immunosensors for the detection of SARS-CoV-2. A field-effect transistor (FET)-based electrochemical immunosensor was developed by coating graphene sheets of the FET with a specific antibody produced against SARS-CoV-2 spike protein. The device could detect the SARS-CoV-2 spike protein with the LOD of 1 fg/mL and 100 fg/mL in PBS and clinical transport medium, respectively. Moreover, in the presence of related viral antigens such as MERS-CoV, this biosensor showed no significant cross-reactivity (57). Earlier, Layqah and Eissa (58) demonstrated an electrochemical immunosensor based on an array of gold nanoparticle modified carbon electrodes for the detection of MERS-CoV. In this biosensor, recombinant spike protein S1 was used as a biomarker for the recognition of MERS-CoV. It could detect the viral antigens within 20 min with a LOD as low as 0.4 pg/mL for H-CoV and 1.0 pg/mL for MERS-CoV. It is anticipated that the principles of this technique could be applied for the detection of related CoV like SARS-CoV-2 with required modification. Recently, Elledge et al. (59) reported a split luciferase antibody biosensor (spLUC) to detect anti-SARS-CoV-2 antibodies in saliva, plasma, whole blood and serum within a very short time. Herein, *S* and *N* proteins were chosen as targets since viral *S* proteins are the epitopes of SARS-CoV-2 antibodies and the *N* protein packages the viral genome into a ribonucleocapsid.

Nevertheless, nucleic acid biosensors are yet to face several challenges. First, to be efficient, the targeted nucleic acid needs to be bio-compatible to the surface of the biosensors. The NA of the virus in clinical specimens (serum, saliva, urine, etc.) are usually encapsulated inside the viral particles. To be detected by the biosensor, the NA must be released from the viral particles through a disruption process usually by thermal or chemical treatment which includes the extra sample pre-treatment step. Second, the purification of nucleic acid is indispensable for the detection of viral genomic NAs in real biological specimens, but it is time consuming and requires extra effort. Furthermore, the viral genome structure is another critical issue associated with the performance of nucleic acid biosensors. Since the single stranded viral genomes are long in structure, they can significantly interfere with sequence-specific probes during the recognition of target NA (55). To overcome the above challenges, further research is needed in designing synthetic single stranded DNA or RNA (aptamer) probe so that intact viral antigens could be recognized directly.

### CRISPR-based Detection

**Figure 3:**
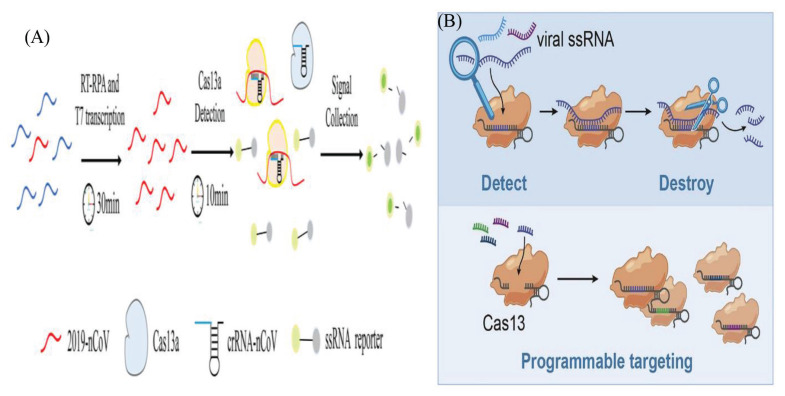
(A) Schematic illustrations of CRISPR-COVID assay. The activation of collateral nuclease activity of Cas proteins occurs following specific binding of gRNA to the Orf1ab gene. Cleaved probes produce fluorescent signal which is then captured and thereby it indicates the presence of SARS-CoV-2 [reprinted from Hou et al. (66) with permission]. (B) Schematic presentation for RNA virus detection using Cas13 [reprinted from Freije et al. (76) with permission]

Clustered-regularly interspaced short palindromic repeats (CRISPR) and CRISPR associated (Cas) protein (CRISPR/Cas) is a rapid, simple and highly promising gene-editing method (60). In this method, a programmable protein becomes attached to the target site by a guide RNA for cleaving the target sequence. The RNA-targeting CRISPR associated enzyme Cas13 has been adapted for quick and portable sensing of nucleic acids (61, 62). The researchers showed that Cas13 can be designed for targeting and destroying the genomes of various mammalian single-stranded RNA viruses (Figure 3). They developed a platform known as SHERLOCK (specific high-sensitivity enzymatic reporter unlocking) which integrated isothermal preamplification with Cas13 for the detection of single RNA or DNA molecules (63). Recently, CRISPR/Cas system has been proposed by Broughton et al. (64) for rapid detection of CoV. Zhang et al. (65) successfully detected SARS-CoV-2 through CRISPR/Cas13 based technique using SHER-LOCK technology. Furthermore, Hou et al. (66) developed and evaluated CRISPR/Cas13a-based diagnostic for COVID-19 and compared it to sequencing-based metagenomic and RT-qPCR-based assays. They found that CRISPR-based technique demonstrated a high sensitivity level (of about single copy) and was highly specific without showing cross reaction with related pathogens. It gives results within only 40 min requiring no expensive thermo-cyclers and thus offers a suitable alternative to the conventional RT-qPCR assay. The technique showed 100% specificity while the PCR assay showed efficiency in 90.4% (47/52) of the positive cases. Guo et al. (67) also established a SARS-CoV-2 detection protocol based on the platform-CDetection (Cas12b mediated DNA detection). They found the detection limit of CASdetec for SARS-CoV-2 pseudovirus down to 1 × 10^4^ copies/mL and observed no cross-reactivity with other endemic human CoVs.

### Microarray-based Detection

Gene microarray is a high throughput technology that can be used for the detection of SARS-CoV-2. The microarray approach consists of two main steps: i) the formation of a specific probe and ii) the generation of targeted cDNA fragments. The microarray assay was successfully used for the detection of CoV (68). Shi et al. (69) developed a 60mer oligonucleotide microarray system targeting TOR2 sequence and successfully detected SARS-CoV-2 in clinical samples. After designing the probes covering the whole genome of the CoV strain, they were immobilised on the microarray surface. RNA was extracted from sample and reverse transcribed into cDNA and cDNAs were fragmented through restriction display (RD) technique. DNA fragments were labeled with Cy5-universal primer through PCR and then hybridisation was completed. Later, a non-fluorescent cheaper, low-density oligonucleotide array was developed to detect the whole CoV genus that was as sensitive as real-time RT-PCR (70). Recently, Hardick et al. (71) developed a portable mobile analysis platform (MAP) based on the microarray chip for CoV detection.

### Aptamer-based Detection

Aptamers, one of the novel DNA receptors, are small-sized, single-stranded artificial DNA or RNA molecules (having 10–100 nucleotides) that can bind and detect various nucleic acid and non-nucleic acid molecules (72). In recent years, viral proteins recognising aptamers have been employed for the rapid detection of viruses including influenza virus (H1N1, H3N2, H5N1 and H9N2) (73) and Zika virus (74). Recently, Chen et al. (75) developed a novel ssDNA aptamer for rapid detection of SARS-CoV-2 *N* protein. They investigated the binding affinity of three aptamers to SARS-CoV-2 *N* protein by Performing the enzyme-linked aptamer binding assay (ELAA) and found that they successfully bound to SARS-CoV-2 *N* protein with high affinity. Moreover, *N* protein of SARS-CoV-2 showed very low similarity (16%–38%) with *N* protein from other five commonly known human CoVs except for SARS-CoV. Thus, aptamer-based biosensors may have enormous potentials for early and rapid diagnosis of COVID-19.

#### Prospects and Challenges

Until now, RT-PCR assays are considered the gold standard for COVID-19 diagnostics. Three important issues are involved with RT-PCR assay. First, PCR reagent kits are not always available to meet the increasing demand. Second, community hospitals at remote and rural areas lack the laboratory infrastructure of PCR setup to accommodate high sample throughput. Lastly, RT-PCR-based detection depends on the presence of detectable SARS-CoV-2 in the collected sample. In case of asymptomatic patient who had been previously infected but now recovered, PCR fails to identify this prior infection and thus control measures would not be possible. Another significant concern is the vulnerability of RNA genomes to degradation by a variety of activities, including damage from environmental factors. Every day, testing laboratories are currently experiencing increased sample load. SARS-CoV-2 viral RNA is degraded due to delays in specimen processing and insufficient storage of suspected samples in laboratories. Viral lysis buffers from RNA extraction kits play a potential role in stabilizing RNA. Currently, researchers have investigated the stability of SARS-CoV-2 RNA in viral lysis buffer at different temperatures and time periods and observed that viral lysis buffer renders samples non-infectious and allows SARS-CoV-2 RNA to be stabilised for up to 48 h even at room temperature. This enables laboratories to maintain a continuous sample processing and testing workflow without compromising timeliness.

The use of antibody-based diagnostics is not suitable to detect active diseases since they give a positive result if the antigens find binding antibodies that might have generated during earlier infection. Although, currently, COVID-19 diagnosis is dominated by PCR and antibody-based techniques, alternative technologies such as LAMP, RT-LAMP, biosensor-based assays, microarray, CRISPR, aptamer-based and other POC systems etc. are now considered promising and may strike the diagnostic market of COVID-19 soon. LAMP can detect small numbers of DNA or RNA templates within a very short time, but the need for high temperature still limits its applicability. Nevertheless, the approach is newer than RT-qPCR and lack enough research background behind it. Biosensor-based assay, although offers rapid, easy and POC detection, however, the targeted nucleic acid needs to be bio-compatible to the surface of the biosensor which is a challenge in its development. Moreover, most cannot achieve multiple detection. The high cost of microarray system inevitably limits its widespread application in the detection of SARS-COV-2. Aptamers suffer from cross reactivity and lack of standardised protocols. To achieve maximum benefit in mass scale diagnostics, biosensors as well as other POC approaches should be further improved. Technologies like barcoding, and microfluidic assays should be further developed to make them plug-and-play systems to implement in an outbreak situation. The combination of diagnostics and smartphones may offer improved efficiency for greater communication and surveillance.

## Conclusion

Although a number of reliable techniques are available to diagnose symptomatic patients in well set up laboratories, remarkable gaps still exist in screening asymptomatic persons during the incubation phase, as well as in exact determination of live viral shedding during the period of convalescence to have decisions to terminate patient isolation. To date, considerable efforts and extensive research endeavors are made worldwide to develop rapid and simple technologies for use in SARS-CoV-2 detection eliminating the shortcomings of the existing methods. To get more benefit in practical applications, some methods are often combined to minimise the limitations of single approach. With the rapid advancement of novel technologies, it is anticipated that in near future there will be more efficient, rapid and low-cost on-site detection methods which would provide scientists/clinicians with more choices.

## Figures and Tables

**Table 1:** Existing SARS-CoV-2 detection techniques and their key features

Techniques	Major Features	Advantages	Limitations	References
RT-qPCR	The target RNA is reverse transcribed to cDNA by reverse transcriptase enzyme followed by amplification of generated cDNA through PCR using specific primers, probes and Taq DNA polymerase enzyme. During the amplification process, probe is cleaved by DNA polymerase due to its 5′ endonuclease activity thereby releasing fluorescent dye which generates a detectable signal.	Can detect current infections of disease. Considered as gold standard for SARS-COV-2 detection. Highly specific, sensitive and reliable. No cross reactivity with related viruses. Highly stable DNA biomarkers are used.	Expensive and complex. Needs sophisticated instruments and expertise to handle. Requires long time. Complicated to design of probe, selection of fluorescence and optimisation.	Chu et al. (21) Chan et al. (22) Corman et al. (23)
Loop-mediated isothermal amplification (LAMP)	A one-tube and fixed temperature method for the amplification of NA involving DNA polymerase and 4- to 6-primers for annealing six unique locations on the target genome. In a 4-primer procedure, two are inner primers and two are outer primers. The amplified DNA is detected based on turbidity, colour change or fluorescence intensity.	Simple, rapid, specific and cost-effective. Does not require different temperature zones as required in PCR. May be a promising method for in-field detection to detect SARS-CoV-2. Involves less expensive instruments, or even simple incubators or water baths. Compared to PCR, sturdier, and less prone to inhibiting substances. Samples can be used without any prior purification. Generally, produces more DNA than PCR.	Less versatile than PCR Inconvenient for multiplexing and quantifying. Optimisation of primers and reaction parameters are the main challenges. Internal control cannot be included, so assay needs to be duplicated.	Jiang et al. (31) Park et al. (32) Yang et al. (27)
Viral genome sequencing	Allows determination of the order of four nucleotide bases present in the entire genome of an organism and thus enables to identify alterations in genes, associations with diseases and phenotypes, as well as identify probable drug targets.	The most comprehensive approach for the detection of the viral NAs important for disease diagnosis. Reflects any probable mutation of virulence gene during viral spread and thus provides useful information for further epidemiological studies of diseases like COVID-19. Potential automation may be done. Data acquisition methods may be applied to any species without significant adaptation (customising).	Not appropriate for regular and large-scale testing. Not recognised as a useful analytical tool, as it is relatively costly, time consuming, complicated and difficult to prepare sample and analyse data. Labour intensive. Requires technical expertise to perform and interpret data. Needs high informatics capacity and special software.	Liu et al. (6) Zhang (40)
ELISA	Immunological technique based on antigen-antibody interaction to detect antigen or antibody in a sample. The antibody generated in response to antigen is conjugated with an enzyme which reacts with colorless substrate to produce a coloured product.	Simple and easy to perform. Highly sensitive and specific. Can be used successfully for SARS-CoV-2 detection by using IgM and IgG antibodies. Applicable for both qualitative and quantitative measurements.	Expensive and labour-intensive to produce antibody. High probability of false positive or negative results. Antibody, being protein, is less stable.	Xiang et al. (48) Guo et al. (44)
LFA	A paper -based technique that involves the migration of an antigen, or antigen–antibody complexes, through a solid support, (i.e. filter paper, nitrocellulose film or agarose) Generally, a labeled antibody is allowed to bind with the unknown antigen. The resulting antigen–antibody complex then migrates under the influence of capillary action through the support. Then, if antigen is present, a second antibody embedded in the solid support detects the complexes.	User-friendly, cheap, and easily produced POC approach that may be promising for in-field detection of SARS-CoV-2. May also be developed by incorporating nucleic acid testing. Rapid, giving result within 5 min–30 min. Allows both detection and quantification of analytes in complex matrices. Portable detection device. Low costs and easy to develop. Suitable for applications in multiple fields.	For single use and suffers from poor detection. sensitivity as compared to RT-PCR. Less suitable for accurate screening of COVID-19 patients.	Li et al. (52) Grant et al. (53)
Biosensors	Short-length single-stranded DNA probe is immobilised on to the surface of the signal transducer. Specific target is hybridised with the probe DNA resulting changes in optical or electrochemical properties which can be measured by the signal intensity.	Offer rapid and POC detection A suitable alternative for rapid detection, real-time analysis and constant monitoring of COVID 19. Portable, very simple and easy to handle. compatible with miniaturised diagnostic devices No significant cross-reactivity Low-cost Highly sensitive	Most cannot achieve multiple detection. For NA based biosensor, pure DNA sample is required. To be efficient, the targeted nucleic acid needs to be bio-compatible to the surface of the biosensors which is a challenge in its development. NA purification step is time consuming and requires extra effort.	Qiu et al. (56) Layqah and Eissa (58)
Microarray	Involves specific probe sequences immobilized in an array format on a solid support and the target fragments attached with fluorescent marker are allowed to bind with immobilised probes. This hybridisation allows to release fluorescent signals and detection is performed based on the signal.	A high throughput technology. Suitable for the detection of virus with a high mutation rate. The diagnostic ratio can be increased while monitoring the viral genes activities during disease progression at the same time. May be developed for POC diagnostic application for coronavirus detection. Can detect a higher number of DNA fragments synchronously.	Expensive and difficult to perform. The quality and amount of RNA remains a major challenge. False microarray data can be generated from degraded mRNA.	Shi et al. (69) Hardick et al. (71)
CRISPR-based detection	A programmable protein becomes attached to the target site by a guide RNA for cleaving the target sequence. A platform known as SHERLOCK integrates isothermal preamplification with Cas13 protein for the detection of single RNA or DNA molecules of the respective virus.	Rapid, sensitive, less expensive. More precise than other genome editing approaches. Can be suitably developed for rapid detection of coronavirus. No need of thermal cycler.	Complex, and laborious. Need expertise to perform.	Broughton et al. (64) Zhang et al. (65)
Aptamer-based detection	Short, single-stranded oligonucleotides that can bind and detect various nucleic and non-nucleic acid molecules with high affinity and specificity and thus help detect any markers of viral infection.	Specific binding capacity to a broad range of molecules (target analytes) with high affinity. Discriminates infected host cells from uninfected ones or active from inactive virus. Rapid detection capability. Enormous potentials for early and rapid diagnosis of COVID-19.	The rapid degradation of aptamers (especially RNA aptamers) by nucleases in biological media seriously limits their practical application. Regardless of their high specificity, they suffer from cross reactivity and can also bind to molecules with a similar structure. Lack of standardised protocols.	Chen et al. (68) Zou et al. (73) Lee and Zeng (74)

**Table 2:** The reported RT-qPCR assays for SARS-CoV-2 detection

Detection techniques	Target gene	Forward primer (5′-3′)	Reversed primer (5′-3′)	Probe (5′-3′)	Reporter/Quencher dyes	References
TaqMan RT-qPCR	ORF1b	TGGGGYTTTACRGGTAACCT (Y=C/T, R=A/G)	AACRCGCTTAACAAAGCACTC (R=A/G)	TAGTTGTGATGCWATCATGACTAG (W=A/T)	FAM/ZEN-IBFQ	Chu et al. (21)
	*N*	TAATCAGACAAGGAACTGATTA	CGAAGGTGTGACTTCCATG	GCAAATTGTGCAATTTGCGG	FAM/ZEN-IBFQ	
TaqMan RT-qPCR	*N*	GCGTTCTTCGGAATGTCG	TTGGATCTTTGTCATCCAATTTG	AACGTGGTTGACCTACACAGST	FAM/ lABkFQ	Chan et al. (22)
	*S*	CCTACTAAATTAAATGATCTCTGCTTTACT	CAAGCTATAACGCAGCCTGTA	CGCTCCAGGGCAAACTGGAAAG	HEX/ lABkFQ	
	RdRp/ Helicase	CGCATACAGTCTTRCAGGCT	GTGTGATGTTGAWATGACATGGTC	TTAAGATGTGGTGCTTGCATACGTAGAC	FAM/ lABkFQ	
TaqMan RT-qPCR	RdRP	GTGARATGGTCATGTGTGGCGG	CARATGTTAAASACACTATTAGCATA	CAGGTGGAACCTCATCAGGAGATGC CCAGGTGGWACRTCATCMGGTGATGC	FAM/BBQ	Corman et al. (23)
	*E*	ACAGGTACGTTAATAGTTAATAGCGT	ATATTGCAGCAGTACGCACACA	ACACTAGCCATCCTTACTGCGCTTCG	FAM/BBQ	
	*N*	CACATTGGCACCCGCAATC	GAGGAACGAGAAGAGGCTTG	ACTTCCTCAAGGAACAACATTGCCA	FAM/BBQ	
TaqMan RT-qPCR	*N1*	GAC CCC AAA ATC AGC GAA AT	TCTGGTTACTGCCAGTTG AATCTG	ACC CCG CAT TAC GTT TGG TGG ACC	FAM/BHQ1	CDC (77)
	*N2*	TTA CAA ACA TTG GCC GCA AA	GCG CGA CAT TCC GAA GAA	ACA ATT TGC CCC CAG CGC TTC AG	FAM/BHQ1	
	*N3*	GGG AGC CTT GAA TAC ACC AAA A	TGT AGC ACG ATT GCA GCA TTG	AYC ACA TTG GCA CCC GCA ATC CTG	FAM/ BHQ1	
SYBR Green RT-qPCR	nsp2	ATGCATTTGCATCAGAGGCT	TTGTTATAGCGGCCTTCTGT	-	SYBR Green	Yip et al. (24)
